# Cost-effectiveness of Human Papillomavirus Vaccination in the United States

**DOI:** 10.3201/eid1402.070499

**Published:** 2008-02

**Authors:** Harrell W. Chesson, Donatus U. Ekwueme, Mona Saraiya, Lauri E. Markowitz

**Affiliations:** * Centers for Disease Control and Prevention, Atlanta, Georgia, USA

**Keywords:** human papillomavirus, vaccine, cost-effectiveness, research

## Abstract

We describe a simplified model, based on the current economic and health effects of human papillomavirus (HPV), to estimate the cost-effectiveness of HPV vaccination of 12-year-old girls in the United States. Under base-case parameter values, the estimated cost per quality-adjusted life year gained by vaccination in the context of current cervical cancer screening practices in the United States ranged from $3,906 to $14,723 (2005 US dollars), depending on factors such as whether herd immunity effects were assumed; the types of HPV targeted by the vaccine; and whether the benefits of preventing anal, vaginal, vulvar, and oropharyngeal cancers were included. The results of our simplified model were consistent with published studies based on more complex models when key assumptions were similar. This consistency is reassuring because models of varying complexity will be essential tools for policy makers in the development of optimal HPV vaccination strategies.

## Abstract 2

Results of a simplified model were consistent with published studies based on more complex models when key assumptions were similar.

In 2000, the Institute of Medicine (IOM) published a report listing 26 candidate vaccines that potentially could be developed and licensed in the first 2 decades of the 21st century (*1*). Included in this list was a candidate vaccine for human papillomavirus (HPV), a virus that can cause cervical and other anogenital cancers, genital warts, and other adverse health outcomes (*1*–*5*). For example, in the United States, HPV types 16 and 18 cause ≈70% of cervical cancer, 80% of anal cancer, and 30% of vaginal and vulvar cancers (*2*–*5*). Furthermore, HPV types 6 and 11 cause >90% of cases of anogenital warts (*5*,*6*). The economic costs of HPV-related genital warts and cervical disease, including screening to prevent cervical cancer, are estimated to be at least $4 billion annually in the United States (*7*,*8*).

In June 2006, the US Food and Drug Administration approved a quadrivalent (HPV 6, 11, 16, 18) vaccine (Gardasil, manufactured by Merck & Co., Inc. [Whitehouse Station, NJ, USA]) for use in girls and women 9–26 years of age (*5*). The efficacy of this vaccine is almost 100% if given to young women before sexual exposure (*3*,*5*,*9*). Also in June 2006, the US Advisory Committee on Immunization Practices recommended routine HPV vaccination for girls 11–12 years of age (*3*). The vaccine series can be initiated in girls as young as 9 years, and catch-up vaccination is recommended for girls and young women of ages 13–26 years who have not received the HPV vaccine previously or who have not completed the full vaccine series (*3*).

In anticipation of the approval of new HPV vaccines, several studies have been conducted to estimate the potential cost-effectiveness of HPV vaccination in the United States in terms of the cost per quality-adjusted life year (QALY) saved (*1*,*9*–*13*). With 1 exception (*1*), these studies applied a Markov model, a decision model, a dynamic transmission model, or a combination thereof (see Dasbach et al. [*14*] for a review of HPV models). To complement these existing studies, we developed a simplified model to estimate the cost-effectiveness of adding HPV vaccination of 12-year-old girls to existing cervical cancer screening practices in the United States. Our approach was similar to that used by IOM (*1*) in that we estimated the potential benefits of HPV vaccination based on current, age-specific incidence rates of HPV-related outcomes. Additionally, our analysis extended the IOM approach to reflect a more current understanding of the vaccine’s characteristics and to include the potential benefits of preventing HPV-related anal, vaginal, vulvar, and oropharyngeal cancers.

## Methods

Similar to the IOM approach, we used spreadsheet software to build an incidence-based model of the health and economic effects of HPV-related health outcomes in the absence of HPV vaccination. We then examined how these effects might change over time because of HPV vaccination, based on factors such as the number of 12-year-old girls vaccinated each year and vaccine efficacy. We adopted a societal perspective and included all direct medical costs (2005 US$) and benefits regardless of who incurred the costs or received the benefits (*15*,*16*). The study question we addressed was “What is the cost per QALY gained by adding vaccination of 12-year-old girls to existing cervical cancer screening practices in the United States?”

### Population Model

A hypothetical population of persons 12–99 years of age was created as follows. First, the number of 12-year-old girls was based on recent sex-specific population estimates (*17*). The number of 13-year-old girls was calculated as the product of the number of 12-year-olds and the probability of survival (using recent mortality data) from age 12 years to age 13 years. The number of 14-year-old girls and the number of persons of all subsequent ages through 99 years were calculated in an analogous manner. We assumed that the number of 12-year-olds each year was constant over time so that the age distribution of the population was constant over time as well.

### Vaccine Coverage, Efficacy, and Costs

We assumed the HPV vaccine would be administered to 12-year-old girls starting in year 1 and continuing through year 100. We assumed that vaccinated girls would receive the full vaccine series (3 doses) before age 13 years. Vaccination coverage (the percentage of 12-year-old girls vaccinated) was assumed to increase linearly for the first 5 years to 70% and to remain at 70% thereafter (*9*). Vaccination efficacy was assumed to be 100%, on the basis of trials showing high efficacy of prophylactic HPV vaccines against persistent infection and vaccine type–specific cervical intraepithelial neoplasia (CIN) grades 2 and 3 (*3*,*18*–*21*). The duration of vaccine protection was assumed to be lifelong, and the cost of vaccination was set to $360 per series (*9*).

### Adverse Health Outcomes Averted by Vaccination

We examined the following HPV-related health outcomes: cervical cancer; CIN grades 1, 2, and 3; genital warts; and, in some analyses, anal, vaginal, vulvar, and selected oropharyngeal cancers. The age-specific incidence rates of the HPV-related health outcomes were used to estimate the potential reduction in these outcomes that could be obtained through vaccination.

Age-specific cancer incidence rates were derived from 2003 population-based cancer registries that participate in the Centers for Disease Control and Prevention’s National Program of Cancer Registries (NPCR) and the National Cancer Institute’s Surveillance, Epidemiology, and End Results Program (SEER) (*22*,*23*). Together, the 2 cancer registries covered ≈96% of the US population in 2003 (*22*). The cancer incidence rates we applied were conservative because we included only certain morphology (histology) codes, which limited cervical cancers to cervical carcinomas (squamous cell, adenocarcinoma, adenosquamous, and other carcinoma) and which limited all other noncervical cancers to squamous cell carcinomas only (*24*). We did not include in situ cancers from the cancer registries. We limited oropharyngeal cancers to selected sites most commonly associated with HPV (base of tongue, tonsillar, and other oropharyngeal sites as described in the Technical Appendix, (*24*).

Age-specific incidence rates of CIN grades 1, 2, and 3, and prevalence rates of genital warts were based on estimates obtained from the literature (*25*,*26*). We used prevalence estimates for genital warts because age-specific incidence estimates were not available Technical Appendix.

### Cervical Cancer Screening

The incidence rates of CIN and cervical cancers that we applied in our model are those that arise in the context of current cervical cancer screening and sexually transmitted disease prevention activities in the United States. Because these prevention activities are reflected in the incidence rates of CIN and cervical cancer that we applied in our model, no information about these prevention activities (e.g., coverage and frequency of cervical cancer screening) was required in our analysis.

### Costs Averted and QALYs Saved by Vaccination

The cervical cancer treatment costs averted by vaccination were calculated each year by multiplying the age-specific number of cervical cancer cases averted by the vaccine in that year by the estimated cost per case of cervical cancer, Technical Appendix. The number of QALYs saved by preventing cervical cancer was calculated for each year by multiplying the age-specific number of cervical cancer cases averted by the vaccine in that year by the estimated age-specific number of QALYs lost per case of cervical cancer, Technical Appendix. For other health outcomes (other cancers, CIN 1, CIN 2, CIN 3, and genital warts), the treatment costs averted and QALYs saved by vaccination were estimated in an analogous manner.

### Age-specific Estimates of Direct Medical Costs and QALYs Lost per Adverse Health Outcome

The estimated direct medical cost per case of cervical cancer and other HPV-related health outcomes was based on several sources (*7*,*10*,*12*,*26*–*35*). The age-specific estimates of the discounted number of QALYs lost per case of an HPV-related heath outcome (e.g., cervical cancer) were based on published estimates of the quality of life without these adverse health outcomes (*36*) and the estimated reduction in quality of life associated with the HPV-related health outcome (*1*,*10*,*12*,*37*) (Technical Appendix).

### Incremental Cost per QALY Gained

Vaccination costs, averted treatment costs, and the number of QALYs saved were calculated for each year over a 100-year period, discounted to present value by using an annual discount rate of 3% (*9*). The incremental cost per QALY gained by adding vaccination to existing cervical cancer screening was calculated as the net cost of vaccination divided by the number of QALYs gained by adding vaccination to existing screening, where the net cost of vaccination is the cost of vaccination minus the treatment costs averted by adding vaccination to existing screening (*16*).

### Herd Immunity Scenario

To examine how the estimated cost-effectiveness of vaccination might change if the benefits of herd immunity were included, we assumed an additional effect of the vaccine on nonvaccinated persons, including a reduction in genital warts in men. The Technical Appendix, provides details of the methods and assumptions used to estimate these additional benefits.

### Cohort Model

To make our results more comparable to Markov models of an age cohort, we modified our population model to examine the benefits of vaccination of a single cohort of 12-year-old girls over time. Vaccination costs were incurred in the first year only, and the benefits of vaccinating the 12-year-old cohort were calculated through age 99 years. Because Markov models of age cohorts typically do not include transmission dynamics, we did not consider the potential benefits of herd immunity in the cohort model.

### Base Case Analyses

Using base-case parameter values (Technical Appendix), we estimated the cost-effectiveness of HPV vaccination by using 12 variations of the model. These 12 variations consisted of 4 permutations (including vs. excluding the noncervical cancers and including vs. excluding the benefits of preventing HPV types 6 and 11) of 3 model versions (population model with and without herd immunity, cohort model without herd immunity).

### Sensitivity Analyses

We performed sensitivity analyses to examine how changes in the base-case parameter values influenced the estimated cost-effectiveness of vaccination. We first examined how the cost-effectiveness estimates of the population model’s herd immunity scenario changed when assumptions about the degree of the effect of herd immunity were changed. The remainder of the sensitivity analyses focused on the population model of the quadrivalent HPV vaccine without the adjustment for herd immunity.

We performed 1-way sensitivity analyses in which we varied 1 set of parameter values while holding other parameters at their base-case values. The parameters we varied included the cost of the vaccine series ($300, $490), vaccine efficacy (95%, 99%), the cost per case of all HPV-related health outcomes (±25% of their base-case values); the discount rate (0%, 5%); the time horizon over which vaccination costs and benefits were assumed to accrue (25 years, 50 years); the incidence rates of health outcomes (±25% of their base-case values for CIN 1, CIN 2, CIN 3, and genital warts, and the lower and upper bound ranges of the 95% confidence interval from the NPCR and SEER data for cancers); the percentage of each health outcome attributable to HPV vaccine types (±20% of their base-case values); and the number of lost QALYs associated with each HPV outcome. We manipulated the last number by varying the reduction in quality of life (±50% of the base-case values) associated with all HPV-related health outcomes and by varying the stage-specific survival probabilities for HPV-related cancers (±2 standard errors). We also performed multiway sensitivity analyses by varying >2 sets of these parameter values simultaneously.

The parameters that were varied in the sensitivity analyses comprised almost all of the parameters in the model. Exceptions included duration of vaccine protection (which is difficult to modify in our model without sacrificing the simplicity of our approach), vaccine coverage (which does not affect our results except when herd immunity is assumed), and other parameters such as age-specific death rates, which are not subject to considerable uncertainty.

### Comparison to Previous Cost-Effectiveness Studies

We compared our results with previously published estimates of the cost-effectiveness of HPV vaccination. To do so, we modified the parameter inputs to match as closely as possible several key attributes of the models applied in these previous studies (Technical Appendix).

## Results

Under base-case parameter values, the estimated cost per QALY gained by adding vaccination of 12-year-old girls to existing cervical cancer screening was $3,906–$14,723, depending on the type of model applied (cohort vs. population), whether herd immunity effects were assumed, the types of HPV targeted by the vaccine (bivalent vs. quadrivalent), and whether the benefits of preventing other cancers in addition to cervical cancer were included (Table 1). If all other factors were equal, the estimated cost per QALY gained by vaccination was lower when herd immunity effects were assumed, when protection against HPV types 6 and 11 (rather than just HPV types 16 and 18) was included, and when the benefits of preventing other cancers in addition to cervical cancer were included.

**Table 1:** Estimated cost per QALY gained by adding routine HPV vaccination of 12-y-old girls to existing cervical cancer screening in the United States*

Parameter	Population model		Cohort model; no herd immunity, $US
	No herd immunity, $US	Herd immunity, $US	
Excluding anal, vaginal, vulvar, and oropharyngeal cancers			
Vaccine targets HPV types 6,11,16,18	10,294	5,336	8,593
Vaccine targets HPV types 16,18	14,723	10,318	12,562
Including anal, vaginal, vulvar, and orophayngeal cancers†			
Vaccine targets HPV types 6,11,16,18	8,137	3,906	6,430
Vaccine targets HPV types 16,18	11,602	7,848	9,471

*When applying base-case parameter values to 12 model variations. QALY, quality-adjusted life year; HPV, human papillomavirus. †The oropharyngeal cancer sites we included were base of tongue, tonsillar, and other sites as described in the Technical Appendix.

Prevention of HPV-related health outcomes resulted in averted treatment costs and QALYs saved. For example, in the population model of the quadrivalent vaccine (when herd immunity benefits and the benefits of preventing cancers other than cervical were excluded), reductions in CIN, cervical cancer, and genital warts accounted for ≈70%, 19%, and 12% of the averted costs, respectively, and ≈33%, 54%, and 13% of the saved QALYs, respectively.

### Sensitivity Analyses

The cost-effectiveness ratios did not change substantially when we modified the assumptions in the population model about the effect of herd immunity. When varying the effect of herd immunity, the cost per QALY gained by vaccination was $3,423–$7,596 for the quadrivalent vaccine and $8,549–$12,354 for the bivalent vaccine, when the benefits of preventing cancers other than cervical were excluded (results not shown).

In the 1-way sensitivity analyses of the population model (excluding assumed herd immunity effects), the discount rate and the time horizon had the greatest effect on the estimated cost per QALY gained (Table 2). When the discount rate was varied from 0% to 5%, the cost per QALY gained ranged from $675 to $24,901 (and from <$0 to $21,966 when other cancers in addition to cervical cancer were excluded). When the time horizon was varied from 25 to 50 years (rather than the base-case value of 100 years), the cost per QALY gained ranged from $21,600 to $81,786 (and from $19,943 to $81,398 when other cancers in addition to cervical cancer were included). Changes in the other sets of parameter values (such as costs and QALYs associated with HPV-related health outcomes) also affected the results, but to a lesser degree than changes in the discount rate and time horizon (Table 2). In the multiway sensitivity analyses, simultaneously changing 2 sets of parameter values resulted in estimated costs per QALY gained of <$0 to $4,606 when parameter values more favorable to vaccination were applied and estimated costs per QALY gained of $17,825 to $36,503 when parameter values less favorable to vaccination were applied (Table 3).

**Table 2:** One-way sensitivity analyses: estimated cost per QALY gained by adding routine vaccination of 12-y-old girls to existing cervical cancer screening in the United States*

Parameter or parameter set varied	Values applied in sensitivity analysis	Cost/QALY gained	
		Excluding anal, vaginal, vulvar, oropharyngeal cancers, $US	Including anal, vaginal, vulvar, oropharyngeal cancers, $US
None	NA	10,294	8,137
Vaccine cost per series (base case = $360)	$300, $490	5,811–20,009	4,237–16,587
Vaccine efficacy (base case = 100%)	95%, 99%	10,566–11,710	8,374–9,369
Cost of cervical cancer, CIN 1–CIN 3, genital warts*	Base case ±25%	6,142–14,446	4,332–11,953
Reduction in quality of life due to HPV-related health outcomes	Base case ±50%†	7,720–15,519	6,141–12,135
Incidence rates of cervical cancer, CIN 1–CIN 3, genital warts‡	Base case ±25%†	6,999–16,333	5,181–13,379
% of health outcomes attributable to HPV vaccine types	Base case ±20%	6,014–17,020	4,400–13,987
Discount rate (base case = 3%)	0%, 5%	675–24,901	<0–21,966
Time horizon (base case = 100 y)	25 y, 50 y	21,600–81,786	19,943–81,398

*When key parameter values were varied in the population model of quadrivalent HPV vaccine (excluding herd immunity). QALY, quality-adjusted life year; HPV, human papillomavirus; NA, not applicable; CIN, cervical intraepithelial neoplasia. †See text and Technical Appendix for details. ‡And, when applicable, anal, vaginal, vulvar, and oropharyngeal cancers.

**Table 3:** Multiway sensitivity analyses: estimated cost per QALY gained by adding routine vaccination of 12-y-old girls to existing cervical cancer screening in the United States*†

Parameter or parameter set varied	Cost per QALY gained	
	Excluding anal, vaginal, vulvar cancers, $US.	Including anal, vaginal, vulvar cancers, $US
Higher cost per case and larger reduction in quality of life for all HPV-related health outcomes	4,606	3,262
Lower cost per case and smaller reduction in quality of life for all HPV-related health outcomes	21,779	17,825
Discount rate = 0%; time horizon = 100 y	675	<0
Discount rate = 5%; time horizon = 50 y	36,503	34,539
Higher percentage of health outcomes attributable to HPV vaccine types; higher incidence of HPV-related health outcomes	3,815	1,882
Lower percentage of health outcomes attributable to HPV vaccine types; lower incidence of HPV-related health outcomes	24,250	20,265
All variables above (best-case scenario)	<0	<0
All variables above (worst-case scenario)	122,976	115,896

*When key parameter values were simultaneously varied in the population model of quadrivalent HPV vaccine (excluding herd immunity). QALY, quality-adjusted life year; HPV, human papillomavirus; †The lower and upper bound ranges were the same as described in the1-way sensitivity analyses, except for the time horizon, which was varied from 50 y to 100 y.

In the best and worst case scenarios (when all 6 selected sets of parameters were set to values more favorable and less favorable to vaccination, respectively), the cost per QALY gained was <$0 and $122,976, respectively (<$0 and $115,896 when including other cancers in addition to cervical cancer) (Table 3). However, much of the variation in the best and worst case scenarios was attributable to changes in the discount rate and the time horizon. For example, when the worst case scenario was modified to include a discount rate of 3% (rather than 5%), the estimated cost per QALY gained (when the benefits of preventing cancers other than cervical were excluded) was ≈$75,000 when applying a 50-year time horizon and $41,000 when applying a 100-year time horizon (results not shown).

### Comparison with Previous Cost-Effectiveness Studies

Estimates from the simplified model were quite consistent with published estimates (Table 4). The absolute difference between the estimated cost per QALY gained by vaccination as estimated by our simplified model and as estimated by the more complex models did not exceed $4,000.

**Table 4:** Summary of previously published models and estimates of the cost per QALY gained by adding routine HPV vaccination of 12-y-old girls to existing cervical cancer screening in the United States*†

Variable	Goldie et al. 2004 (*10*)	Sanders and Taira 2003 (*11*)	Taira et al. 2004 (*13*)	Elbasha et al. 2007 (*9*)
Key assumptions in published models				
Target of HPV vaccine	HPV 16,18	High risk HPV types	HPV 16,18	HPV 6,11,16,18
Efficacy of vaccine	90%	75%	90%	100%‡
Vaccine cost per series	$393	$300	$300 + $100 booster	$360
Base year of $US	2002	2001	2001	2005
Estimated cost per QALY of vaccination				
Published model estimate	$24,300	$12,700§	$14,600	$3,000
Simplified model estimate	$20,600	$8,700	$17,100	$5,300

*QALY, quality-adjusted life year; HPV, human papillomavirus. †In all comparisons, the simplified model was modified (as necessary) so that the assumptions regarding the target of the HPV vaccine, vaccine efficacy and cost, vaccine duration of protection (except in the comparison to Taira and colleagues [*13*], as noted in the Technical Appendix, and the base year of US$ were consistent with the published models (Technical Appendix). The simplified model estimate was based on the cohort model in the comparisons with the findings of Goldie et al. (*10*) and Sanders and Taira (*11*) and was based on the population model (assuming transmission effects) in the comparison with the estimates of Taira and colleagues (*13*) and Elbasha and colleagues (*9*). ‡Elbasha and colleagues (*9*) assumed 90% protection against infection with HPV and 100% protection against HPV-related disease.§To enhance comparability, the published estimate from Sanders and Taira (*11*) was based on their sensitivity analyses when assuming lifetime duration of vaccination, not their base-case estimate of $22,800 when 10-y vaccine duration of protection was assumed.

## Discussion

We developed a simple model to estimate the cost-effectiveness of HPV vaccination in the context of current cervical cancer screening in the United States. We found that the cost per QALY gained by adding routine vaccination of 12-year-old girls to existing screening practices ranged from $3,906 to $14,723 under base-case parameter values (depending on the model version we applied) and ranged from <$0 (cost-saving) to $122,976 in the sensitivity analyses when several key parameter values were varied. Our results were consistent with results of published studies based on more complex models, particularly when key assumptions (e.g., vaccine duration, efficacy, and cost) were similar.

The simplicity of our approach offers advantages and disadvantages. The main advantage is that it requires substantially fewer assumptions than the more complex Markov and transmission models. For example, there is no need to model the probability of HPV acquisition, the possible progression from HPV infection to disease, the mixing of sex partners, the probability of HPV transmission, and so forth. There also is no need to model cervical cancer screening and sexually transmitted disease prevention activities because these activities are reflected in the incidence rates of HPV-related health outcomes that we applied.

Because we do not model cervical cancer screening directly, however, we are unable to use our model to examine how changes in cervical cancer–screening strategies can affect the cost-effectiveness of HPV vaccination, and vice versa. For example, HPV vaccination is expected to reduce the positive predictive value of abnormal Papanicolaou (Pap) test results (*38*). However, our analysis did not include the loss in quality of life attributable to the initial distress associated with receiving an abnormal Pap result (*39*), regardless of whether it is a false positive. This omission of the lost QALYs due to abnormal Pap test results underestimates the benefits of HPV vaccination because vaccination is expected to offer moderate reductions in the number of abnormal Pap results overall (*38*,*40*). Future changes in screening strategies, such as delayed screening, could also possibly improve the cost-effectiveness of HPV vaccination (*12*).

Another disadvantage of our approach is that it offers only a rough approximation of the cost-effectiveness of HPV vaccination and is not suitable for examining strategies such as vaccination of boys and men. In addition, although many of the parameter values and assumptions in our model can be modified with ease, changing the assumption of lifelong duration of protection or examining vaccination at older ages would require the incorporation of assumptions about the incidence and natural history of HPV to account for the probability of acquiring HPV (before vaccination or after vaccine immunity wanes) and the subsequent probability of adverse HPV-attributable health outcomes. However, we can address the issue of waning immunity by assigning a higher cost per vaccination series (as in the sensitivity analyses) to reflect the cost of a booster.

Another limitation of our approach is the uncertainty in the key parameter values, such as the cost and loss in quality of life associated with HPV-related health outcomes, the percentage of health outcomes attributable to each type of HPV targeted by the vaccine, and the incidence of CIN and genital warts. However, our results were fairly robust in response to changes in these key parameter values. For example, when simultaneously varying the costs of HPV-related health outcomes and the loss in QALYs associated with HPV-related health outcomes, we found that the estimated cost per QALY gained by vaccination ranged from $3,262 to $21,779.

Our adjustments for the effect of herd immunity were arbitrary; we simply assumed an additional effect of vaccination in the nonvaccinated population. However, our results did not vary substantially (in absolute terms) when the assumed effect of herd immunity was varied. For example, the estimated cost per QALY gained by quadrivalent vaccination (including herd immunity and excluding the benefits of preventing cancers other than cervical) was $5,336 in the base case and ranged from $3,423 to $7,596 when the adjustments for the effects of herd immunity (including the impact on genital warts in males) were varied. We also note that the benefits to nonvaccinated persons were assumed to occur only in nonvaccinated persons of similar ages to those vaccinated. This restriction may have understated the potential benefits of herd immunity.

Our analysis did not address all of the potential costs and benefits of vaccination. For example, the cost-effectiveness estimates would have been more favorable to vaccination if we had included the potential for cross-protection against high-risk HPV types besides 16 and 18 (*21*); the prevention of anal, vaginal, and vulvar cancer precursor lesions (as demonstrated in the supplemental analysis in the Technical Appendix; the prevention of other cancers not included in this analysis (such as anal cancer and oropharyngeal cancers in male patients); and the prevention of other HPV-related health outcomes such as recurrent respiratory papillomatosis. Conversely, the cost-effectiveness estimates would have been less favorable to vaccination if we had included the potential for HPV type replacement (i.e., an increase in HPV types not protected against by vaccination), waning immunity, and the possible costs and loss in quality of life associated with adverse side effects of vaccination.

A key finding from this analysis was that the choice of discount rate and time horizon has a substantial influence on the estimated cost-effectiveness of vaccination. Because the costs of HPV vaccination begin to accrue immediately but the full benefits of vaccination are not realized for many years, the cost-effectiveness of vaccination becomes less favorable when higher discount rates are applied or when shorter time horizons are examined.

Another key finding was that the potential benefits of preventing anal, vaginal, vulvar, and oropharyngeal cancers offer nontrivial improvements in the estimated cost-effectiveness of HPV vaccination. The inclusion of these additional benefits decreased the cost per QALY gained by vaccination by ≈$2,200 (or 21%) in the population model (without herd immunity), by ≈$1,400 (or 27%) in the population model (with herd immunity), and by ≈$2,200 (or 25%) in the cohort model. Future studies that develop better estimates of the cost and loss in quality of life associated with these cancers could more accurately estimate the effects of these additional benefits on the cost-effectiveness of HPV vaccination. Despite the limitations discussed above, our simplified model provides useful estimates of cost-effectiveness of HPV vaccination in the United States. Our results were consistent with previous studies based on more complex models. This consistency is reassuring because models of various degrees of complexity will be essential tools for policy makers in the development of optimal HPV vaccination strategies.

## Supplementary Material

Technical Appendix The Cost-Effectiveness of HPV Vaccination in the United States: Estimates from a Simplified Model.
