# Supplementary material for: Cost-effectiveness of Human Papillomavirus Vaccination in the United States
Source: Emerg Infect Dis. 2008 Feb;14(2):244–51. doi: 10.3201/eid1402.070499 (PMC2600200; doi:10.3201/eid1402.070499)
Supplement: Technical Appendix — The Cost-Effectiveness of HPV Vaccination in the United States: Estimates from a Simplified Model. [file 07-0499_Techapp-s1.pdf]

The Cost-Effectiveness of HPV Vaccination in the United States: Estimates from a Simplified Model

Methods

We adopted a societal perspective and included all direct medical costs and benefits regardless of who incurred the costs or received the benefits (1,2). Indirect costs such as the lost productivity associated with cervical cancer mortality (3) and direct nonmedical costs such as patent travel time were not included in this analysis because previous studies of HPV vaccination cost-effectiveness focused primarily on direct medical costs and because estimates of indirect and direct nonmedical costs are not available for all HPV-related health outcomes. Costs were expressed in 2005 U.S. dollars, except where noted in the comparisons with previous studies.

Vaccine coverage, efficacy and costs

Assumptions regarding vaccine characteristics are summarized in Appendix Table 1. We assumed the HPV vaccine would be administered to 12-year-old girls starting in year 1 and continuing through year 100. We assumed vaccinated girls would receive the full vaccine series (three doses) before age 13 years. Vaccination coverage (the percentage of 12-year-old girls vaccinated) in years 5 through 100 was 70% in the base case (4). We assumed vaccination coverage increased linearly, such that coverage rates in years 1, 2, 3, and 4 were 0.2, 0.4, 0.6, and 0.8 times (respectively) the coverage rates in years 5 through 100 (4). Vaccination efficacy was assumed to be 100%, based on trials showing high efficacy of prophylactic HPV vaccines against persistent infection and vaccine type-specific CIN 2 and 3 (5–9). The duration of vaccine protection was assumed to be lifelong, and the cost of vaccination was set to $360 per series (4).

Adverse health outcomes averted by vaccination

We examined the following HPV-related health outcomes: cervical cancer; cervical intraepithelial neoplasias (CIN) grades 1, 2, or 3; genital warts; and in some analyses, anal, vaginal, vulvar, and selected oropharyngeal cancers. Estimates of the age-specific incidence rates of these health outcomes (Appendix Table 2) in the absence of vaccination were used to estimate the potential reduction in these outcomes that could be obtained through vaccination. For example, the number of cervical cancer cases averted by the vaccine in a given year t for a given age group i was estimated as: $R_i\,(P_i/100{,}000)(A_{16} + A_{18})EC_{i,t}$, where R_i is the rate of cervical

cancer (per 100,000) in age group i, P_i is the number of females in age group i, A_{16} and A_{18} are the percentages of cervical cancer attributable to HPV 16 and HPV 18, respectively, E is vaccine efficacy, and $C_{i,t}$ is the coverage of vaccination in age group i in year t (the percentage of persons in age group i in year t who were vaccinated at age 12). The number of cases of other health outcomes (other cancers, CIN 1, CIN 2, CIN 3, and genital warts) averted by vaccination was estimated in a manner analogous to that for cervical cancer.

The estimated percentage of cervical cancer attributable to HPV 16 and 18 (as well as the fraction of other health outcomes attributable to various HPV types) was based on several sources (10–26)(Appendix Table 3). We assumed that the proportion of cancers attributable to HPV types 16 and 18, respectively, was 76% and 7% for anal cancer, 28% and 4% for vaginal cancer, 29% and 3% for vulvar cancer, and 31% and 1% for the selected oropharyngeal cancers we included in this analysis (Appendix Table 3). These attributable proportions for anal, vaginal, and vulvar cancers were selected such that the proportion attributable to HPV 16 and 18 was consistent with a recent review of the burden of HPV-related cancers (19), and the impact of HPV 16 relative to HPV 18 was consistent with a range of published estimates (11–18,20). The attributable proportions for the selected oropharyngeal cancers were based on a review indicating HPV prevalence of 35.6% in oropharyngeal squamous cell carcinomas (SCCs), with HPV 16 and HPV 18 accounting for 86.7% and 2.8% of the HPV-positive oral SCCs, respectively (26). We assumed that the proportion of CIN 2 attributable to HPV vaccine types was the same as that of CIN 3, as the source study for this information (23) did not provide different estimates for CIN 2 and CIN 3. This assumption may have caused a slight overestimation of the role of HPV 16 and 18 in CIN 2 and a slight underestimation of the role of HPV 16 and 18 in CIN 3.

Age-specific incidence rates of anal, cervical, vaginal, vulvar, and oropharyngeal cancers were derived from 2003 population-based cancer registries that participate in the Centers for Disease Control and Prevention's National Program of Cancer Registries (NPCR) and the National Cancer Institute's Surveillance, Epidemiology, and End Results (SEER) Program (27,28). Together, the two cancer registries covered ≈96% of the United States population in 2003 (27). SEER*Stat software was used to calculate incidence rates and 95% confidence intervals (29). Because the estimates of the percentage of each cancer attributable to HPV vaccine types (described above) were based on overall site and not on morphology, we expect

that our application of these estimated attributable fractions will be conservative given that the cancer incidence rates we applied were limited (based on morphology) to those more likely to be HPV-related (30). That is, we limited cervical cancers to include cervical carcinomas (squamous cell, adenocarcinomas, adenosquamous, and other carcinoma) and we limited all other non-cervical cancers to include squamous cell carcinomas only (30). Further, we limited oropharyngeal cancers to selected sites more commonly associated with HPV (base of tongue, tonsillar, and other oropharyngeal sites), using the following International Classification of Diseases for Oncology (ICD-O-3) codes: 019, 024, 090, 091, 098, 099, 142, 028, 102, 108, 109, 140, and 148 (30,31).

Age-specific incidence rates of CIN grades 1, 2, and 3, and prevalence rates of genital warts were based on estimates obtained from the literature (32,33). Prevalence rates of genital warts in persons aged 65 years and older were adjusted as follows. Rates for persons aged 65 to 69 years were assumed to be as estimated by Insinga and colleagues (33) for ages 65 years and older. The decrease in prevalence from the 60- to 64-year age group to the 65- to 69-year age group was applied to all subsequent age groups, such that genital warts prevalence after age 65 years declined steadily with age. We used prevalence estimates for genital warts because age-specific incidence estimates were not available. In the study from which the prevalence estimates were obtained (33), the mean duration of genital warts episodes was ≈3 months, suggesting that these annual prevalence rates may be similar to annual incidence rates. In the absence of vaccination, our model predicts ≈486,000 new cases of genital warts each year, which is consistent with published estimates ranging from 250,000 to 1,000,000 (34).

Costs averted and QALYs saved by vaccination

The cervical cancer treatment costs averted by vaccination were calculated each year by multiplying the age-specific number of cervical cancer cases averted by the vaccine in that year (as described above) by the estimated cost per case of cervical cancer. The estimated cost per case of cervical cancer and other HPV-related health outcomes was based on several sources (33–45)(Appendix Table 1). The cost per case of genital warts we applied was based three published estimates (33–35) and was reduced by 25% to account for the possibility that genital warts cases might go untreated (34). We estimated the cost per case of vaginal and vulvar cancers under the assumption that the ratio of these costs to the cost of anal cancer (43) was

similar to the ratio of these costs as reported in a study of the potential costs averted by HPV vaccination in British Columbia (44). The cost of oropharyngeal cancers was based on average Medicare payments among persons with head and neck cancers in the first year of illness minus the average Medicare payments among matched comparison patients (45).

The number of QALYs saved by preventing cervical cancer was calculated for each year by multiplying the age-specific number of cervical cancer cases averted by the vaccine in that year by the estimated age-specific number of QALYs lost per case of cervical cancer (described below and summarized in Appendix Table 4). For other health outcomes (other cancers, CIN 1, CIN 2, CIN 3, and genital warts), the treatment costs averted and QALYs saved by vaccination were estimated in an analogous manner.

Age-specific estimates of QALYs lost per adverse health outcome

The age-specific estimates of the discounted number of QALYs lost per case of cervical cancer and other cancers, CIN 1, CIN 2, CIN 3, and genital warts were based on published estimates of the quality of life without adverse these health outcomes (46) and the estimated reduction in quality of life associated with these HPV-related health outcomes (38,41,47,48), as described below.

Cervical cancer was assumed to lead to one of six outcomes, based on three possible stages at diagnosis (local, regional, or distant) and two possible survival outcomes (survival or death). For survivors, the relative loss in quality of life associated with treatment and followup was 0.27 for 4 months for local cancer, 0.37 for 3 years for regional cancer, and 0.45 for 3 years for distant cancer; with subsequent lifelong relative losses in quality of life of 0.07 for local cancer, 0.1 for regional cancer, and 0.24 for distant cancer. For non-survivors, a relative loss in quality of life (0.36 for local cancer, 0.41 for regional cancer, and 0.45 for distant cancer) was assumed for 3 years, followed by death. The quality weights described above, and the estimated durations of these reductions in quality of life, were based on previously published estimates (41,47,48). Based on SEER data, the distributions of cervical cancer stage at diagnosis (local, regional, distant) we applied were: 64%, 29%, and 7%, respectively, for women under 50 years of age and 40%, 46%, and 14%, respectively, for women 50 years of age and older. For women 41–49 years of age, we used a linear combination of these two distributions to allow for a gradual change with age in the distribution of cancer stage at diagnosis. We applied probabilities

of survival for local, regional, and distant cervical cancer of 0.94, 0.64, and 0.20, respectively, for women under 50 years of age and 0.87, 0.50, and 0.09, respectively, for women 50 years of age and older.

We used a similar approach to calculate the lost QALYs attributable to other cancers. For each cancer, we applied the same stage-specific quality weights as for cervical cancer, but applied cancer-specific distributions of the stage at diagnosis (local, regional, or distant) and cancer-specific, stage-specific survival probabilities. The distributions of stage at diagnosis (local, regional, or distant) we applied were: 56%, 35%, and 9% for anal cancer; 38%, 37%, and 25% for vaginal cancer; 60%, 35%, and 5% for vulvar cancer; and 17%, 69%, and 13% for oropharyngeal cancers. These distributions were applied to all age groups. The survival probabilities we applied for local, regional, and distant cancer were: 0.86, 0.65, and 0.26 for anal cancer; 0.79, 0.41, and 0.32 for vaginal cancer; 0.91, 0.55, 0.23 for vulvar cancer; and 0.58, 0.51, and 0.25 for oropharyngeal cancers, based on 5-year survival probabilities obtained from SEER data. For vaginal cancer diagnosed in the regional stage, the survival probability we applied (0.41) reflects the upper bound value suggested by the SEER data. We used this higher value so that the relative change in survival probability across cancer stages (local, regional, and distant) for vaginal cancer was consistent with that of the other cancers.

CIN 1 was assumed to cause a relative loss in quality of life of 0.03 for 18 months, with no effect thereafter (47). CIN 2 was assumed to cause a relative loss in quality of life of 0.07 for 18 months, with no effect thereafter, based on estimates of the impact of CIN 1 (47) and the relative impact of CIN 2 to CIN 1 on quality of life (38). CIN 3 was assumed to cause a relative loss in quality of life of 0.2 for 4 months and 0.03 for 2 years, and with no effect thereafter (47).

For genital warts in females, the relative loss of quality of life and the duration of such loss were assumed to be one of the following four scenarios: 0.05 loss for 3 months, 0.1 loss for 6 months, 0.15 loss for 3 months, or 0.15 loss for 6 months, with probability 0.475, 0.475, 0.025, and 0.025, respectively (47). For genital warts in males, the relative loss of quality of life and the duration of such loss were assumed to be one of the following four scenarios: 0.1 loss for 3 months, 0.1 loss for 6 months, 0.15 loss for 3 months, or 0.15 loss for 6 months, with probability 0.475, 0.475, 0.025, and 0.025, respectively (47).

We used these estimates of the impact of HPV-related health outcomes (cervical cancer, CIN 1, 2, and 3, and genital warts) on quality of life to estimate the QALYs lost per case of each health outcome. For example, as noted above, CIN 1 was assumed to cause a relative loss in quality of life of 0.03 for 18 months. For a 20-year-old female, the number of lost QALYs associated with CIN 1 was calculated as $0.03(Q_{20}/2) + 0.03(Q_{21}S_{20})/(1+r)$, where Q_t denotes the expected quality of life for a female at age t years in the absence of genital warts, r is the discount rate, and S_{20} is the probability that a 20-year-old female would survive to at least age 21 years. The first 6 months of lost quality of life were assumed to occur at age 20 (the Q_{20} term is divided by 2 to reflect 6 months of lost quality of life rather than 1 year) and the final 12 months of lost quality of life were assumed to occur at age 21. The lost quality of life at age 21 was adjusted to reflect the probability of survival to age 21 and discounted to present value at the time of onset of the health outcome (in this case, age 20). For other ages, and for other health outcomes (cervical cancer and other cancers, CIN 2, CIN 3, and genital warts), the age-specific estimates of the QALYs lost per health outcome were calculated in an analogous manner. The resulting age-specific estimates of the number of lost QALYs associated with these health outcomes, under the assumptions described above, are summarized in Appendix Table 4.

Cost per QALY gained

Vaccination costs, averted treatment costs, and the number of QALYs saved were calculated for each year over a 100-year time period, discounted to present value using an annual discount rate of 3% (49). The cost per QALY gained by vaccination was calculated as (V-A)/Q, where V is the cost of vaccination, A is the averted treatment costs due to vaccination, and Q is the number of QALYs saved due to vaccination (50).

Herd immunity scenario

To examine how the estimated cost-effectiveness of vaccination might change if the benefits of herd immunity were included, we assumed an additional impact of the vaccine on non-vaccinated persons, including a reduction in genital warts in males. For each health outcome in females (cervical and other cancers, CIN 1, CIN 2, CIN 3, and genital warts), we assumed that the number of cases averted in non-vaccinated females in a given age group would be equal to the number of cases averted in vaccinated females in that age group multiplied by the percentage of females in that age group not vaccinated multiplied by an adjustment factor F ($0 \leq F \leq 1$). For

example, the number of cases of cervical cancer averted through herd immunity in age group i in year t was calculated as $R_i(P_i/100{,}000)(A_{16} + A_{18})EC_{i,t}(1-C_{i,t})F$.

Vaccination of females would be expected to reduce genital warts in males as well. In the herd immunity scenario, we assumed the percentage reduction in genital warts in males was M ($0<M<1$) times the overall percentage reduction in genital warts in females.

We applied 0.5 as the base case value of M, which is consistent with, but slightly more conservative, than the 0.56 relative reduction in male HPV prevalence (as compared to the reduction in female HPV prevalence) predicted by a population-level transmission model of female HPV vaccination (51). We also applied 0.5 as the base case value of F, due to a lack of available estimates for this parameter value. The implication of this assumption is as follows. With 70% coverage and 100% efficacy, the direct impact of vaccination in our model is a 70% reduction in health outcomes attributable to the HPV vaccine types in females. When herd immunity benefits are included, using the adjustment factor $F = 0.5$, the population-level impact would be an 80.5% reduction in health outcomes attributable to the HPV vaccine types in females. The uncertainty associated with the adjustment factors F and M is addressed later in the sensitivity analyses.

Cohort model

To make our results more comparable to Markov models of an age cohort, we modified our population model to examine the benefits of vaccination of a single cohort of 12-year-old girls over time. Vaccination costs were incurred in the first year only, and the benefits of vaccinating the 12-year-old cohort were calculated through age 99 years. The benefits of vaccination (averted treatment costs and QALYs saved) were calculated as in the main analysis described above. Because Markov models of age cohorts typically do not include transmission dynamics, we did not consider the potential benefits of herd immunity in the cohort model.

Base case parameter values

Base case parameter values are summarized in Appendix Tables 1–4. Using these base case parameter values, we estimated the cost-effectiveness of HPV vaccination under 12 variations of the model (Appendix Table 5). These 12 variations consisted of 4 permutations (including versus excluding anal, vaginal, vulvar, and oropharyngeal cancers; and including

versus excluding the benefits of preventing HPV types 6 and 11) of 3 model versions (the population model with and without herd immunity; and the cohort model without herd immunity).

Sensitivity analyses

We performed sensitivity analyses to examine how changes in the base case parameter values influenced the estimated cost-effectiveness of vaccination. We first examined how the estimated cost-effectiveness estimates of the population model's herd immunity scenarios (model variations 5 and 6 in Appendix Table 5, which exclude anal, vaginal, vulvar, and oropharyngeal cancers) changed under varying assumptions about the impact of herd immunity (F) and the relative impact (M) of female vaccination on genital warts in males compared to females (*F* = M = 0.25, *F* = M = 0.75). The remainder of the sensitivity analyses focused on the population model of the quadrivalent HPV vaccine without the adjustment for herd immunity (model variations 1 and 3 in Appendix Table 5).

We performed one-way sensitivity analyses in which we varied one set of parameter values while holding other parameters at their base case values. The parameters varied included the cost of the vaccine series ($300, $490); vaccine efficacy (95%, 99%); the cost per case of all HPV-related health outcomes (±25% of their base case values); the discount rate (0%, 5%); the time horizon over which vaccination costs and benefits were assumed to accrue (25 years, 50 years); the incidence rates of health outcomes (±25% of their base case values for CIN1, CIN 2, CIN 3, and genital warts, and the lower and upper bound ranges of the 95% confidence interval from the NPCR and SEER data for cancers); the percentage of each health outcome attributable to HPV vaccine types (±20% of their base case values); and the loss in quality of life associated with each HPV-related outcome, which was manipulated by varying the reduction in quality of life (±50% of their base case values) associated with all HPV-related health outcomes and by varying the survival probabilities for HPV-related cancers (±2 standard errors). We also performed multi-way sensitivity analyses by varying two or more sets of these parameter values simultaneously.

Comparison with previous cost-effectiveness studies

To compare our results with previously published estimates, we modified the parameter inputs to match as closely as possible several key attributes of the models applied in these

previous studies. Specifically, we used our cohort analyses when comparing our results to that of published Markov models and used our population model with assumed herd immunity effects when comparing our results to those of transmission models, and we closely followed the other models in their assumptions regarding vaccine price, efficacy, coverage, and duration of protection; base year in which costs were reported; and HPV types targeted by the vaccine (bivalent or quadrivalent).

In our comparison to the Markov model of Goldie et al. (41), we applied our cohort model, adjusted two parameter values (vaccine efficacy = 0.9, vaccine cost = $393), and calculated the cost per QALY gained in 2002 U.S. dollars. We focused only on the benefits of preventing HPV 16 and 18, and excluded herd immunity effects and the benefits of preventing anal, vaginal, vulvar, and oropharyngeal cancers.

In our comparison to the Markov model of Sanders and Taira (52), we applied our cohort model, set vaccine cost to $300, and calculated the cost-per-QALY gained in 2001 U.S. dollars. Sanders and Taira examined a vaccine with 75% efficacy against high risk HPV types. To mirror this assumption in our model, we assumed 100% efficacy against HPV 16 and 18 but changed the percent of cervical cancer attributable to HPV 16 and 18 from 70% to 75%. We adjusted the percentage of CIN 1, CIN 2, and CIN 3 attributable to HPV 16 and 18 by the same proportion. We focused only on the benefits of preventing HPV 16 and 18, and excluded herd immunity effects and the benefits of preventing anal, vaginal, vulvar, and oropharyngeal cancers. Finally, to make the comparison more valid, we compared our results to Sanders and Taira's estimated cost per QALY of $12,700 when assuming lifetime duration of protection, rather than their base case estimate of $22,800 when assuming 10 years duration of vaccine protection.

In our comparison to the transmission model of Taira et al. (53), we applied our population model (with herd immunity included), adjusted two parameter values (vaccine efficacy = 0.9, vaccine cost = $374), and calculated the cost per QALY gained in 2001 U.S. dollars. The $374 cost was chosen to reflect the $300 cost of the vaccine series and the discounted cost of the $100 booster required 10 years after the initial series as assumed by Taira and colleagues. We were unable to match the assumption of Taira and colleagues of waning vaccine protection 10 years after the booster. Thus, in this comparison, the initial vaccine series and booster shot were assumed to provide lifetime protection in our simplified model as opposed

to 20 years protection in the model by Taira and colleagues. We focused only on the benefits of preventing HPV 16 and 18 and excluded the benefits of preventing anal, vaginal, vulvar, and oropharyngeal cancers.

In our comparison to the transmission model of Elbasha et al. (4), we applied our population model and calculated the cost per QALY gained in 2005 U.S. dollars. We included the benefits of protection against HPV 6,11,16 and 18 and the benefits of herd immunity, but excluded the benefits of preventing anal, vaginal, vulvar, and oropharyngeal cancers.

Because our simplified approach does not directly incorporate cervical cancer screening activities (which instead are reflected in the incidence rates of CIN and cervical cancer we applied), we did not compare our model results to those of Kulasingam and Myers (38), who examined the cost-effectiveness of vaccination in the context of various cervical cancer screening strategies.

Additional analysis and results: Anal, vaginal, and vulvar cancer precursor lesions

As a supplement to the main analyses, we also examined how the estimated cost-effectiveness of vaccination might change when including the potential impact of vaccination on the incidence of anal, vaginal and vulvar cancer precursor lesions. The inclusion of these additional health outcomes is problematic due to the lack of information available on the treatment cost and quality of life impacts of these health outcomes. However, to obtain a rough approximation of the potential impact of the inclusion of these precursor lesions on our results, we estimated the vaccine impact (treatment costs averted and QALYs saved) on anal, vaginal, and vulvar cancer precursor lesions as described below. We note, however, that the anal, vaginal, and vulvar cancer precursor lesions were included only in this supplemental analysis, and were not included in the results reported elsewhere.

The treatment cost averted by preventing anal, vaginal, and vulvar cancer precursor lesions was calculated as $(\theta_1/\theta_2)\times\theta_3\times\sigma$, where θ_1 is the averted costs associated with cervical cancer precursor lesions (CIN 1–3), θ_2 is the averted cost of cervical cancer, θ_3 is the averted cost of anal, vaginal, and vulvar cancers, and σ is an adjustment factor. That is, we assumed the ratio of the averted costs of anal, vaginal, and vulvar cancer precursor lesions to the averted costs of

anal, vaginal, and vulvar cancers would be equal to the ratio of the averted costs of cervical cancer precursor lesions to the averted costs of cervical cancer, multiplied by an adjustment factor (σ). We first used an adjustment factor of 0.5, as the ratio of the cost of precursor lesions to the cost of cancer may be lower for anal, vaginal, and vulvar cancers than for cervical cancer, because screening (which can increase the number of precursor lesions detected and reduce the incidence of cancer) is more common for cervical cancer than for anal, vaginal, or vulvar cancers. We also applied an adjustment factor of 1.0 to examine the sensitivity of the results to this adjustment. The number of QALYs saved by preventing anal, vaginal, and vulvar cancer precursor lesions was estimated in an analogous manner.

As reported in the main text, when applying the population model without assuming herd immunity, the estimated cost per QALY averted by a quadrivalent vaccine was $8,137 when including anal, vaginal, vulvar, and oropharyngeal cancers. When anal, vaginal and vulvar cancer precursor lesions were also included as described above, the estimated cost per QALY was $6,754 (when $\sigma = 0.5$) and $5,447 (when $\sigma = 1.0$).

References

1. Gold MR, Siegel JE, Russell LB, Weinstein MC, eds. Cost-effectiveness in health and medicine. Report of the Panel on Cost-Effectiveness in Health and Medicine. New York: Oxford University Press; 1996.

2. Haddix AC, Teutsch SM, Corso PS, eds. Prevention Effectiveness: A Guide to Decision Analysis and Economic Evaluation, 2nd edition. New York: Oxford University Press; 2003.

3. Insinga RP. Annual productivity costs due to cervical cancer mortality in the United States. Womens Health Issues. 2006;16:236–42. Medline

4. Elbasha EH, Dasbach EJ, Insinga RP. Model for assessing human papillomavirus vaccination strategies. Emerg Infect Dis. 2007;13:28–41. Medline

5. Centers for Disease Control and Prevention Quadrivalent human papillomavirus vaccine: Recommendations of the Advisory Committee on Immunization Practices (ACIP). MMWR Recomm Rep. 2007;56(RR-2):1–24.

6. Koutsky LA, Ault KA, Wheeler CM, Brown DR, Barr E, Alvarez FB, et al. A controlled trial of a human papillomavirus type 16 vaccine. N Engl J Med. 2002;347:1645–51. Medline

7. Mao C, Koutsky LA, Ault KA, Wheeler CM, Brown DR, Wiley DJ, et al. Efficacy of human papillomavirus-16 vaccine to prevent cervical intraepithelial neoplasia: a randomized controlled trial. Obstet Gynecol. 2006;107:18–27. Medline

8. Villa LL, Costa RL, Petta CA, Andrade RP, Ault KA, Giuliano AR, et al. Prophylactic quadrivalent human papillomavirus (types 6, 11, 16, and 18) L1 virus-like particle vaccine in young women: a randomised double-blind placebo-controlled multicentre phase II efficacy trial. Lancet Oncol. 2005;6:271–8. Medline

9. Harper DM, Franco EL, Wheeler CM, Moscicki AB, Romanowski B, Roteli-Martins CM, et al. Sustained efficacy up to 4.5 years of a bivalent L1 virus-like particle vaccine against human papillomavirus types 16 and 18: follow-up from a randomised control trial. Lancet. 2006;367:1247–55. Medline

10. Dunne EF, Markowitz LE. Genital human papillomavirus infection. Clin Infect Dis. 2006;43:624–9. Medline

11. Frisch M, Glimelius B, van den Brule AJ, Wohlfahrt J, Meijer CJ, Walboomers JM, et al. Sexually transmitted infection as a cause of anal cancer. N Engl J Med. 1997;337:1350–8. Medline

12. Daling JR, Madeleine MM, Johnson LG, Schwartz SM, Shera KA, Wurscher MA, et al. Human papillomavirus, smoking, and sexual practices in the etiology of anal cancer. Cancer. 2004;101:270–80. Medline

13. Daling JR, Madeleine MM, Schwartz SM, Shera KA, Carter JJ, McKnight B, et al. A population-based study of squamous cell vaginal cancer: HPV and cofactors. Gynecol Oncol. 2002;84:263–70. Medline

14. Carter JJ, Madeleine MM, Shera K, Schwartz SM, Cushing-Haugen KL, Wipf GC, et al. Human papillomavirus 16 and 18 L1 serology compared across anogenital cancer sites. Cancer Res. 2001;61:1934–40. Medline

15. Madeleine MM, Daling JR, Carter JJ, Wipf GC, Schwartz SM, McKnight B, et al. Cofactors with human papillomavirus in a population-based study of vulvar cancer. J Natl Cancer Inst. 1997;89:1516–23. Medline

16. Koyamatsu Y, Yokoyama M, Nakao Y, Fukuda K, Saito T, Matsukuma K, et al. A comparative analysis of human papillomavirus types 16 and 18 and expression of p53 gene and Ki-67 in cervical, vaginal, and vulvar carcinomas. Gynecol Oncol. 2003;90:547–51. Medline

17. Bjorge T, Engeland A, Luostarinen T, Mork J, Gislefoss RE, Jellum E, et al. Human papillomavirus infection as a risk factor for anal and perianal skin cancer in a prospective study. Br J Cancer. 2002;87:61–4. Medline

18. Iwasawa A, Nieminen P, Lehtinen M, Paavonen J. Human papillomavirus in squamous cell carcinoma of the vulva by polymerase chain reaction. Obstet Gynecol. 1997;89:81–4. Medline

19. Parkin DM, Bray F. Chapter 2: The burden of HPV-related cancers. Vaccine. 2006;24(Suppl 3):S11–25. Medline

20. Holm R, Tanum G, Karlsen F, Nesland JM. Prevalence and physical state of human papillomavirus DNA in anal carcinomas. Mod Pathol. 1994;7:449–53. Medline

21. Bosch FX, de Sanjosé S. Chapter 1: Human papillomavirus and cervical cancer–burden and assessment of causality. J Natl Cancer Inst Monogr. 2003;3–13.

22. Clifford GM, Rana RK, Franceschi S, Smith JS, Gough G, Pimenta JM. Human papillomavirus genotype distribution in low-grade cervical lesions: comparison by geographic region and with cervical cancer. Cancer Epidemiol Biomarkers Prev. 2005;14:1157–64. Medline

23. Clifford GM, Smith JS, Aguado T, Franceschi S. Comparison of HPV type distribution in high-grade cervical lesions and cervical cancer: a meta-analysis. Br J Cancer. 2003;89:101–5. Medline

24. Silverberg MJ, Ahdieh L, Munoz A, Anastos K, Burk RD, Cu-Uvin S, et al. The impact of HIV infection and immunodeficiency on human papillomavirus type 6 or 11 infection and on genital warts. Sex Transm Dis. 2002;29:427–35. Medline

25. Greer CE, Wheeler CM, Ladner MB, Beutner K, Coyne MY, Liang H, et al. Human papillomavirus (HPV) type distribution and serological response to HPV type 6 virus-like particles in patients with genital warts. J Clin Microbiol. 1995;33:2058–63. Medline

26. Kreimer AR, Clifford GM, Boyle P, Franceschi S. Human papillomavirus types in head and neck squamous cell carcinomas worldwide: a systematic review. Cancer Epidemiol Biomarkers Prev. 2005;14:467–75. Medline

27. U.S. Cancer Statistics Working Group. United States cancer statistics: 2003 incidence and mortality. Atlanta: Department of Health and Human Services, Centers for Disease Control and Prevention, and National Cancer Institute; 2006.

28. Hankey BF, Ries LA, Edwards BK. The surveillance, epidemiology, and end results program: a national resource. Cancer Epidemiol Biomarkers Prev. 1999;8:1117–21. Medline

29. SEER*Stat software [computer program]. Version 6.3.5. Bethesda, MD: National Cancer Institute; 2006.

30. Watson M, Saraiya M, Weir H, et al. Leading partnerships to assess the burden of HPV-related cancers. 2007 CDC Cancer Conference, Atlanta, August 13–17, 2007.

31. World Health Organization. International classification of diseases for oncology, third edition. Geneva: World Health Organization; 2000.

32. Insinga RP, Glass AG, Rush BB. Diagnoses and outcomes in cervical cancer screening: A population-based study. Am J Obstet Gynecol. 2004;191:105–13. Medline

33. Insinga RP, Dasbach EJ, Myers ER. The health and economic burden of genital warts in a set of private health plans in the United States. Clin Infect Dis. 2003;36:1397–403. Medline

34. Chesson HW, Blandford JM, Gift TL, Tao G, Irwin KL. The estimated direct medical cost of sexually transmitted diseases among American youth, 2000. Perspect Sex Reprod Health. 2004;36:11–9. Medline

35. Alam M, Stiller M. Direct medical costs for surgical and medical treatment of condylomata acuminata. Arch Dermatol. 2001;137:337–41. Medline

36. Insinga RP, Glass AG, Rush BB. The health care costs of cervical human papillomavirus-related disease. Am J Obstet Gynecol. 2004;191:114–20. Medline

37. Brown AD, Garber AM. Cost-effectiveness of 3 methods to enhance the sensitivity of Papanicolaou testing. JAMA. 1999;281:347–53. Medline

38. Kulasingam SL, Myers ER. Potential health and economic impact of adding a human papillomavirus vaccine to screening programs. JAMA. 2003;290:781–9. Medline

39. Maxwell GL, Carlson JW, Ochoa M, Krivak T, Rose GS, Myers ER. Costs and effectiveness of alternative strategies for cervical cancer screening in military beneficiaries. Obstet Gynecol. 2002;100:740–8. Medline

40. Goldie SJ, Kim JJ, Wright TC. Cost-effectiveness of human papillomavirus DNA testing for cervical cancer screening in women aged 30 years or more. Obstet Gynecol. 2004;103:619–31. Medline

41. Goldie SJ, Kohli M, Grima D, Weinstein MC, Wright TC, Bosch FX, et al. Projected clinical benefits and cost-effectiveness of a human papillomavirus 16/18 vaccine. J Natl Cancer Inst. 2004;96:604–15. Medline

42. Mandelblatt JS, Lawrence WF, Womack SM, Jacobson D, Yi B, Hwang Y, et al. Benefits and costs of using HPV testing to screen for cervical cancer. JAMA. 2002;287:2372–81. Medline

43. Goldie SJ, Kuntz KM, Weinstein MC, Freedberg KA, Palefsky JM. Cost-effectiveness of screening for anal squamous intraepithelial lesions and anal cancer in human immunodeficiency virus-negative homosexual and bisexual men. Am J Med. 2000;108:634–41. Medline

44. BC Cancer Agency, Cancer Prevention Program. A population based HPV immunization program in British Columbia: Background paper. 2006.

45. Lang K, Menzin J, Earle CC, Jacobson J, Hsu M. The economic cost of squamous cell cancer of the head and neck: findings from linked SEER-Medicare data. Arch Otolaryngol Head Neck Surg. 2004;130:1269–75. Medline

46. 38. Gold MR, Franks P, McCoy KI, Fryback DG. Toward consistency in cost-utility analyses: using national measures to create condition-specific values. Med Care. 1998;36:778–92. Medline

47. Institute of Medicine. Vaccines for the 21st century: A tool for decisionmaking. Washington, DC: National Academy of Sciences; 2000.

48. Kim JJ, Wright TC, Goldie SJ. Cost-effectiveness of alternative triage strategies for atypical squamous cells of undetermined significance. JAMA. 2002;287:2382–90. Medline

49. Corso PS, Haddix AC. Time effects. In: Haddix AC, Teutsch SM, Corso PS, eds. Prevention effectiveness: A guide to decision analysis and economic evaluation, 2nd edition. New York: Oxford University Press, 2002:92–102.

50. Gift TL, Haddix AC, Corso PS. Cost-effectiveness analysis. In: Haddix AC, Teutsch SM, Corso PA, eds. Prevention effectiveness. A guide to decision analysis and economic evaluation, 2nd edition. Oxford: Oxford University Press, 2002:156–77.

51. Hughes JP, Garnett GP, Koutsky L. The theoretical population-level impact of a prophylactic human papilloma virus vaccine. Epidemiology. 2002;13:631–9. Medline

52. Sanders GD, Taira AV. Cost-effectiveness of a potential vaccine for human papillomavirus. Emerg Infect Dis. 2003;9:37–48. Medline

53. Taira AV, Neukermans CP, Sanders GD. Evaluating human papillomavirus vaccination programs. Emerg Infect Dis. 2004;10:1915–23. Medline

Table 1: Base case values of vaccine characteristics, treatment costs, and other parameters

Parameter	Base case value	Source
Vaccine characteristics		
Vaccine efficacy	100%	(5–9)
Duration of vaccine protection	Lifetime	(4)
Vaccine coverage	70%	(4)*
Vaccine cost per series	$360	(4)
Treatment cost per case		
Genital warts	$425	(33–35)*
CIN 1	$1,739	(36,38–42)
CIN 2	$3,233	(36,38–42)
CIN 3	$3,671	(36,38–42)
Cervical cancer	$31,120	(37–42)
Anal cancer	$29,206	(43)
Vaginal cancer	$24,837	(43,44)*
Vulvar cancer	$20,468	(43,44)*
Oropharyngeal cancers	$34,098	(45)*
Herd immunity adjustment factors*		
F (females)	0.5	Assumed
M (males)	0.5	Assumed
Other		
Discount rate, annual	3%	(49)
Time horizon	100 y	Assumed

CIN: cervical intraepithelial neoplasia.
Treatment cost estimates reflect the expected, discounted lifetime costs, and were updated to 2005 U.S. dollars using the medical care component of the consumer price index.
*See text for more details.

Table 2: Age specific incidence rates of cervical and other cancers, CIN 1, CIN2, CIN 3, and prevalence rates of genital warts (per 100,000) applied in the model

Age	Cervical cancer	CIN 1	CIN 2	CIN 3	Genital warts (females)	Genital warts (males)	Anal cancer	Vaginal cancer	Vulvar cancer	Oropharyngeal cancers
12–14	0	0	0	0	43	41	0	0	0	0
15–19	0*	160	80	30	287	65	0	0	0*	0
20–24	1.3	510	320	130	620	293	0	0*	0*	0*
25–29	5.8	140	380	410	394	501	0*	0*	0.2	0*
30–34	11.3	240	140	180	265	388	0*	0*	0.4	0.2
35–39	13.5	240	140	180	199	252	0.6	0.2	0.9	0.4
40–44	15.3	120	50	50	139	189	1.5	0.3	1.8	1.0
45–49	13.7	120	50	50	144	128	2.6	0.4	2.2	1.7
50–54	12.6	70	40	10	92	118	3.4	0.6	2.5	2.5
55–59	13.4	70	40	10	86	86	4.3	0.7	2.6	3.5
60–64	12.2	40	10	0	76	100	5.0	0.7	3.3	4.5
65–69	12.3	40	10	0	55	87	4.4	1.6	4.4	5.2
70–74	11.3	20	0	10	40	76	5.6	1.4	5.8	5.2
75–79	10.5	20	0	10	29	66	5.6	1.9	8.0	4.3
80–84	10.7	0	0	0	21	57	5.4	2.3	9.7	4.0
85–89	9.0	0	0	0	15	50	5.5	3.2	11.7	2.9
90–94	9.0	0	0	0	11	43	5.5	3.2	11.7	2.9
95–99	9.0	0	0	0	8	38	5.5	3.2	11.7	2.9

CIN: cervical intraepithelial neoplasia. Cancer incidence rates were obtained from NPCR and SEER data (see appendix text). Oropharyngeal cancer sites included base of tongue, tonsillar, and other sites as described elsewhere in this appendix. The CIN incidence rates and genital warts prevalence rates were obtained from studies by Insinga and colleagues (32,33). The prevalence rates of genital warts in persons over age 65 y were adjusted as described elsewhere in this appendix. We assumed a rate of 0 for CIN in the 12- to 14-y age group.
*Cancer case counts are suppressed and incidence rates are not calculated if fewer than 16 cases. We assumed a rate of 0 in these instances.

Table 3: Estimated percentages of health outcomes attributable to various HPV types

Health outcome	HPV 6,11	HPV 16	HPV 18	Source
Genital warts	90.0%	0%	0%	(10,24,25)
CIN 1	6.3%	19.4%	9.2%	(22)
CIN 2	0%	45.8%	10.0%	(23) *
CIN 3	0%	45.8%	10.0%	(23) *
Cervical cancer	0%	58.0%	12.0%	(10,19,21)
Anal cancer	0%	76.0%	7.0%	(11,12,14,17,19,20) *
Vaginal cancer	0%	28.0%	4.0%	(13,14,16,19) *
Vulvar cancer	0%	29.0%	3.0%	(14–16,18,19) *
Oropharyngeal cancers	0%	31%	1%	(26) *

*See appendix text for more details.

Table 4: Expected number of discounted lifetime quality-adjusted life years (QALYs) lost as a result of HPV-related health outcomes, by age group

Age	Cervical cancer	CIN 1	CIN 2	CIN 3	Genital warts (females)	Genital warts (males)	Anal cancer	Vaginal cancer	Vulvar cancer	Oropharyngeal cancers
12–14	6.6	0.04	0.10	0.12	0.03	0.04	8.1	12.6	7.6	13.8
15–19	6.4	0.04	0.09	0.11	0.03	0.04	7.9	12.2	7.4	13.3
20–24	6.1	0.04	0.09	0.11	0.03	0.04	7.5	11.7	7.1	12.8
25–29	5.8	0.04	0.09	0.11	0.03	0.04	7.2	11.1	6.8	12.2
30–34	5.5	0.04	0.09	0.11	0.03	0.04	6.8	10.5	6.4	11.5
35–39	5.1	0.04	0.09	0.11	0.03	0.03	6.3	9.8	6.0	10.7
40–44	5.4	0.04	0.09	0.11	0.03	0.03	5.8	9.0	5.5	9.8
45–49	6.4	0.04	0.09	0.11	0.03	0.03	5.3	8.2	5.0	8.9
50–54	6.5	0.04	0.09	0.11	0.03	0.03	4.7	7.3	4.5	8.0
55–59	5.7	0.04	0.08	0.10	0.03	0.03	4.2	6.4	3.9	7.0
60–64	4.9	0.03	0.08	0.10	0.03	0.03	3.6	5.5	3.4	6.0
65–69	4.1	0.03	0.08	0.10	0.03	0.03	3.0	4.6	2.8	5.0
70–74	3.2	0.03	0.08	0.09	0.03	0.03	2.4	3.6	2.3	4.0
75–79	2.5	0.03	0.07	0.08	0.02	0.03	1.8	2.8	1.7	3.0
80–84	1.8	0.03	0.07	0.08	0.02	0.03	1.4	2.0	1.3	2.2
85–89	1.3	0.03	0.07	0.08	0.02	0.03	1.0	1.5	0.9	1.6
90–94	1.0	0.03	0.07	0.08	0.02	0.03	0.8	1.1	0.8	1.3
95–99	0.4	0.02	0.06	0.07	0.02	0.03	0.4	0.5	0.3	0.5

CIN: cervical intraepithelial neoplasia. See appendix text for details.

Table 5: Description of 12 model variations estimated using base case parameter values

Variation	Type of model	Herd immunity included?	Anal, vaginal, vulvar, and oropharyngeal cancers included?	Protection against HPV 6–11 included?
1	Population	No	No	Yes
2	Population	No	No	No
3	Population	No	Yes	Yes
4	Population	No	Yes	No
5	Population	Yes	No	Yes
6	Population	Yes	No	No
7	Population	Yes	Yes	Yes
8	Population	Yes	Yes	No
9	Cohort	No	No	Yes
10	Cohort	No	No	No
11	Cohort	No	Yes	Yes
12	Cohort	No	Yes	No
